# Dabigatran-idarucizumab pharmacokinetics-pharmacodynamics in sheep undergoing cardiopulmonary bypass

**DOI:** 10.1177/02676591251406086

**Published:** 2025-12-01

**Authors:** Michael P Eaton, Sergiy M Nadtochiy, Tatsiana Stefanos, Brian J Anderson

**Affiliations:** 1 University of Rochester School of Medicine and Dentistry, Rochester, NY, USA; 2 Department Anesthesiology, 62710 University of Auckland, Auckland¸ New Zealand

**Keywords:** dabigatran, idarucizumab, coagulation, cardiopulmonary bypass, pharmacokinetics, pharmacodynamics, idarucizumab

## Abstract

**Background:**

The effect of the anticoagulant, dabigatran, and its antagonist, idarucizumab, on coagulation remains poorly quantified. There are few pharmacokinetic-pharmacodynamic data available to describe the interaction in humans or animals undergoing cardiopulmonary bypass.

**Methods:**

Six sheep were given intravenous dabigatran infusion while undergoing cardiopulmonary bypass. Blood samples were collected for thromboelastographic reaction time (R-time) and drug assay at 1. 5, 15, 30, 60, 90, and 120 min after starting dabigatran. Further reaction times were measured at 1 min, 5 min, 15 min, 60 min, 4 h and 24 h after initiation of idarucizumab infusion. Plasma dabigatran concentrations, the dabigatran- idarucizumab interaction and R-times were analyzed using an integrated pharmacokinetic-pharmacodynamic model with non-linear mixed effects.

**Results:**

A 2-compartment model described dabigatran pharmacokinetics with a clearance (CL 0.0509 L/min/70 kg), intercompartment clearance (Q 0.229 L/min/70 kg), central volume of distribution (V1 3.89 L/70 kg), peripheral volume of distribution (V2 11.4 L/70 kg). The peripheral volume was 2.25 times larger during bypass. The effect compartment model estimates for an E_MAX_ model using reaction time had an effect site concentration (Ce_50_ 40.8 mg/L) eliciting half of the maximal effect (E_MAX_ 180 min). A potency factor for the antagonist, idarucizumab (EA_50_ 29.9 mg/L), moved the dabigatran response relationship to the left.

**Conclusions:**

Dabigatran reversibly binds to the active site on the thrombin molecule, preventing activation of coagulation factors. Expansion of peripheral volume of distribution of dabigatran was observed during cardiopulmonary bypass, contributing to observed concentrations lower than predicted. A competitive interaction model adequately described dabigatran reversal by idarucizumab. These data and consequent parameter estimates inform future clinical studies in both animals and humans.

## Introduction

Unfractionated heparin is the most commonly used anticoagulant in both cardiopulmonary and extracorporeal circuits. Concerns about heparin induced thrombocytopaenia has spurred examination of direct thrombin inhibitors.^1^ Although bivalirudin and argatroban have no reversal agent available, dabigatran is a potential alternative because its effect is reversable.

Dabigatran is an anticoagulant drug that reversibly binds to the active site of the thrombin molecule; it is classified as a direct thrombin inhibitor. It also reduces thrombin-mediated inhibition of fibrinolysis. Dabigatran anticoagulant effect can be reversed using the monoclonal antibody, idarucizumab. Intravenous idarucizumab immediately decreases unbound dabigatran concentration.^2^ Idarucizumab is an inhibitor that forms complexes with dabigatran to counteract its anticoagulant effect. It binds to dabigatran that is free or bound to thrombin.

Investigation using animal models is a first step used to guide investigation in humans. We have investigated the dabigatran pharmacokinetic-pharmacodynamic relationship in rabbits using a delayed effect compartment model.^3,4^ The effect measure of thromboelastographic reaction time (R-time) was used as a convenient point-of-care whole blood test reflecting inhibition of the tissue factor pathway. Investigation of dabigatran as an anticoagulant in sheep involved establishing dabigatran pharmacokinetics and a concentration-response relationship using R-time.^5^ This enabled dose prediction using the target concentration strategy^6,7^ for anticoagulant investigation of sheep undergoing cardiopulmonary bypass.^8^ The target concentration was determined as 5 mg/L but observed dabigatran concentrations in sheep during cardiopulmonary bypass were lower than anticipated (mean dabigatran concentration 3.5 mg/L).^8^ This current analysis sought to understand why observed concentrations were not as predicted.^8^ This anticoagulant investigation of sheep undergoing cardiopulmonary bypass^8^ also collected a richer data set for interrogation of the dabigatran concentration-response and dabigatran-idarucizumab relationships. An understanding of these relationships informs future clinical studies in both animals and humans undergoing cardiopulmonary bypass.

## Methods

### Animals and materials

Animal data from two studies were combined for analysis. There were 233 observations from 11 sheep available for pharmacokinetic-pharmacodynamic (PKPD) analysis.

*Study 1.* Five sheep were available for study (weights 28.7 kg, 33.8 kg, 33.9 kg, 34.0 kg, 41.8 kg). These sheep did not undergo cardiopulmonary bypass. Dabigatran 4 mg/kg was injected over 1 min immediately after the baseline (time 0) draw. Blood samples for dabigatran assay and R time were taken at 5, 15, 30, 60, 90, and 120 min. Idarucizumab (15 mg/kg) was given over 30 s immediately after the 120 min of dabigatran injection, and additional blood samples were collected at 5, 15, 30, 60, 120, 240, 480 min, and 24 h. All experimental protocols were approved by the Association for Assessment and Accreditation of Laboratory Animal Care-accredited University of Rochester Committee on Animal Resources. Nonlinear mixed models were used to quantify both dabigatran pharmacokinetics and describe a concentration effect relationship using reaction time (R-time) of the thromboelastogram (TEG). This information was used to determine dose that would achieve a target dabigatran concentration of 5 mg/L is a subsequent study involving sheep undergoing cardiopulmonary bypass. Comprehensive details regarding methodology and results have been published.^5^

*Study 2*. Six sheep (weights 27.7, 24.4, 25.1, 24.5, 28.7, 25.5 kg) were subjected to 120 min of CPB after anticoagulation with intravenous dabigatran. Sedation/analgesia were accomplished with intravenous administration of ketamine (4 mg/kg) and midazolam (0.4 mg/kg), while general anesthesia was maintained with 1-3% isoflurane following endotracheal intubation and controlled ventilation. The cardiopulmonary bypass circuit was primed with dabigatran to the target concentration of 5 mg/L. Cannulation was via the right external jugular vein and carotid artery. Bypass commenced after an ACT of over 400 s was achieved. Vacuum-assisted venous drainage was used in every case, with flow rates maintained between 60-80 mL/kg/min for 120 min. The target mean arterial pressure (MAP) was greater than 50 mmHg. The sheep were weaned from bypass, and a reversal agent was administered after hemodynamic stability was assured.

Dabigatran was administered as a loading dose (dabigatran 0.25 mg/kg) followed by an intravenous infusion of 0.0175 mg/kg/min for 30 min and a subsequent infusion dabigatran 0.0075 mg/kg/min for 90 min until the end of bypass (i.e., 120 min). The cardiopulmonary bypass circuit (volume 0.45 L) was primed with dabigatran to the target concentration of 5 mg/L. Plasma was taken for dabigatran assay at 1 min, 5 min, 15 min, 30 min, 60 min, 90 min and 120 min after starting cardiopulmonary bypass. Idarucizumab (IV loading dose 50 mg/kg followed by a maintenance dose of 50 mg/kg infused over 1 h) was administered after cardiopulmonary bypass to reverse anticoagulation. Reaction time (R-time) was monitored during cardiopulmonary bypass (1 min, 5 min, 15 min, 30 min, 60 min, 90 min and 120 min) and after the loading dose of idarucizumab at 15 min. Further samples for R-time were taken after completion of the 1-h idarucizumab infusion at 5 min, 1 h, 6 h and 24 h. Comprehensive details regarding methodology and results have been published.^8^

### Dabigatran pharmacokinetics

Population parameter estimates were obtained using nonlinear mixed effects models (NONMEM 7.5, ICON Development Solutions, MD, USA). These models account for population parameter variability (between subjects) and residual variability (random effects) as well as parameter differences predicted by covariate (fixed) effects. Population parameter variability was described using exponential models, which is equivalent to assuming a log-normal distribution and avoids biologically inappropriate parameter values of zero or less. Residual unidentified variability (RUV) was modeled using both proportional (RUV_PROP_) and additive residual (RUV_ADD_) errors. The ADVAN6 subroutine was used to solve differential equations. NM-TRAN code is available in supplementary material (Supplemental NM-TRAN Code). A simultaneous PPPD method was used for final pharmacodynamic parameter estimates.^9^ Convergence criterion was three significant digits.

*Pharmacokinetics:* A two-compartment (central and peripheral) pharmacokinetic model was fitted to data. The model was parameterized in terms of clearance (CL), between compartment clearance (Q), central volume (V1) and peripheral volume of distribution (V2). An additional effect compartment was linked to the central compartment by a rate constant (keo). That constant was expressed as a half-time (T_1/2_keo = Ln (2)/keo). The pharmacokinetic parameter values were standardized for a body weight of 70 kg (a standard adult human^10^) using allometric models.^11^ This standardization allows comparison of sheep parameter estimates with those reported for human adults^12^:

Pi=PSTD×(WiWSTD)EXP· Fi

where P_i_ is the parameter of the i^th^ subject, W_i_ is the weight of the i^th^ subject and P_STD_ is the parameter of standard weight (W_STD_) of 70 kg. The EXP exponent was 0.75 for clearance and 1 for distribution volumes.^13^

The effect of cardiopulmonary bypass in volumes and clearances was examined by incorporating scaling factors (F_i_) to the structural pharmacokinetic parameters (V1, V2, CL, Q).

### Dabigatran-effect relationship

Dabigatran pharmacodynamics were described using a sigmoidal E_MAX_ model with the R-time as an effect measure. This reaction time (R-time) represents the time until initial fibrin formation after mechanical stress and reflects the ability to generate thrombin. Population parameter estimates were estimated using an effect compartment model, a model valid for situations where there is an apparent temporal displacement between plasma concentration (Cp) and response e.g., neuromuscular blocking drugs.^14^ A rate constant (keo, T_1/2_keo = Ln (2)/keo) links plasma concentration with effect site concentration (Ce).

EffectDABI=E0+EMAX x CeNCe50N+CeN

The parameter E0 is the baseline measure (e.g., R-time), E_MAX_ is the maximum drug effect, Ce_50_ is the effect site concentration eliciting half of E_MAX_ and N is the Hill coefficient describing the steepness of the concentration–response curve^15^

### Dabigatran-idarucizumab interaction

A competitive interaction model was used to describe the effect of idarucizumab on dabigatran R-time.

EffectDABI=E0+EMAX x CeNCe50 Nx (1+CIDAEA50)+CeN

where *C*_*IDA*_ is the plasma concentration of idarucizumab and EA_50_ is a potency measure of the antagonist (idarucizumab). No concentration assays were available for idarucizumab and so these were estimated based on reported parameter estimates in humans (CL 0,0394 L/min, V 6.47 L).^15^

*Quality of fit.* Model selection required an improvement in the NONMEM objective function between nested models, equating to a reduction >3.84 based on a Chi square distribution (α < 0.05). A visual predictive check (VPC), was used to evaluate how well the model predicted the distribution of observed dabigatran concentrations or coagulation measures (R-time). Parameter uncertainty was determined using bootstrap analysis to determine 95% confidence intervals.^16^

## Results

### Pharmacokinetics

**Figure 1.**
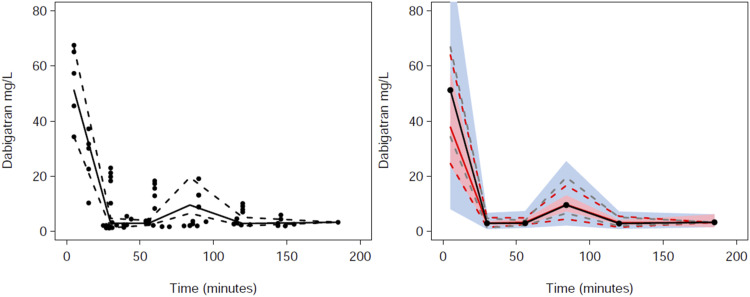
Visual predictive checks (VPC) for dabigatran pharmacokinetics. Plots show median (solid) and 90% intervals (dashed lines). The left hand plot shows all prediction corrected observed dabigatran concentrations. The right hand plot shows prediction corrected percentiles (10%, 50%, and 90%) for observations (grey dashed lines) and predictions (red dashed lines) with 95% confidence intervals for prediction percentiles (median, pink shading; 5th and 95th blue shading).

Pharmacokinetic population parameter estimates for a sheep of standardized weight 70 kg are shown in Table 1. Clearance (0.0509 L/min/70 kg) in this pooled data set was larger than those sheep who did not undergo bypass (0.0453 L/min/70 kg). Use of a scaling factor (F_i_) for the structural peripheral volume of distribution parameter (FV2) was 2.25 while on CPB. Parameter estimates are scaled to a typical human weight of 70 kg for convenience of comparing characteristics between species. This does not change the relationship between size and parameters; it simply changes the scale of the parameter.^12^ The visual predictive check (VPC) is shown in Figure 1.

**Table 1.** Standardized dabigatran population pharmacokinetic parameter estimates. BSV is the between subject parameter variability, SE is the standard error, CI is the confidence interval.

Parameter	Estimate	%BSV	95% CI
CL_STD_ (L/min/70 kg)	0.0509	42.9	0.048, 0.076
V1_STD_ (L/70 kg)	3.89	62.7	3.52, 6.12
Q_STD_ (L/min/70 kg)	0.229	69.5	0.187, 0.325
V2_STD_ (L/70 kg)	11.4	36.6	9.63, 18.9
F_V2_	2.25	-	1.84, 16.3
CL_IDA_ (L/min)	0.0394 FIX	-	-
V_IDA_ (L/kg)	6.47 FIX	-	-
RUV_ADD_ (mg/L)	0.0078	-	0.001, 0.0129
RUV_PROP_ (%)	22.7	-	19.2, 25.9

### Pharmacodynamics

**Figure 2.**
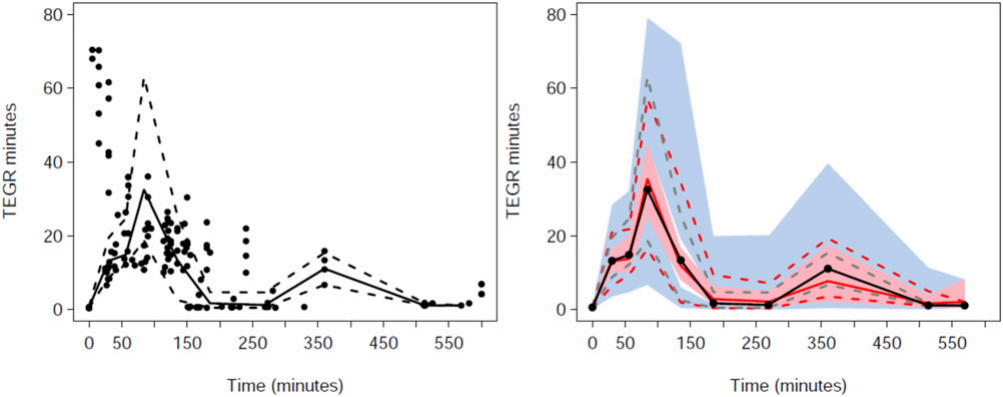
Visual predictive checks (VPC) for the pharmacodynamic reaction time (R) response. Plots show median (solid) and 90% intervals (dashed lines). The left hand plot shows all prediction corrected observed dabigatran concentrations. The right hand plot shows prediction corrected percentiles (10%, 50%, and 90%) for observations (grey dashed lines) and predictions (red dashed lines) with 95% confidence intervals for prediction percentiles (median, pink shading; 5th and 95th blue shading).

**Figure 3.**
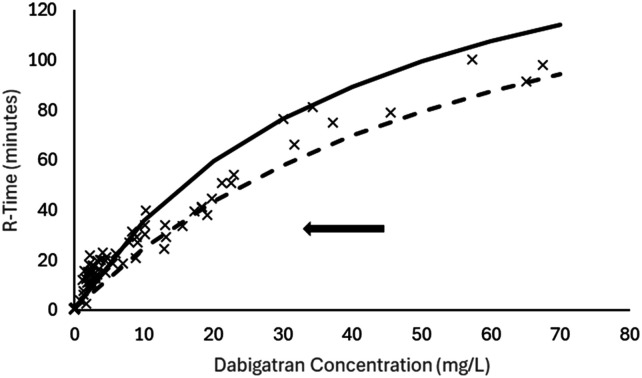
The dabigatran concentration-response relationship reveals a leftward shift in the R-Time response in pooled analysis that included sheep undergoing cardiopulmonary bypass, reflected by a lower EC_50_ 40.8 min compared to those who did not undergo cardiopulmonary bypass (EC50 64.2 min). The population mean for the pooled analysis is shown as a solid line. The population mean for sheep not undergoing bypass is shown as a dashed line. Individual bayesian estimates are shown as x.

Pharmacodynamic population parameter estimates are shown in Table 2. The visual predictive check (VPC) plots for the effect of dabigatran on reaction time (Figure 2) confirmed the adequacy of model predictions with little apparent deviations between model and data. The 90% confidence interval and median for observed data lie within the predicted intervals that were obtained by simulation. The dabigatran-concentration response was more revealing (Figure 3). There was a leftward shift of the R-time response in pooled analysis that included sheep undergoing cardiopulmonary bypass, reflected by a lower EC_50_ 40.8 min compared to those who did not undergo cardiopulmonary bypass (EC_50_ 64.2 min).

**Table 2.** Pharmacodynamic population parameter estimates for reaction times (R).

Parameter	Estimate	BSV%	95%CI
Effect compartment model			
E0 (min)	0.584	15.1	0.469, 0.682
Emax (min)	180 FIX	-	-
Ce_50_ (mg/L)	40.8	19.9	38.2, 49.9
N	1 FIX	-	-
T_1/2_keo (min)	0.011	-	-
EA_50_ (mg/L)	29.9	105.4	19.9, 94.3
RUV_ADD_	0.119	-	0.107, 0.167
RUV_PROP_ (%)	53.5	-	48.3, 74.5

### Impact of idarucizumab on reaction time

**Figure 4.**
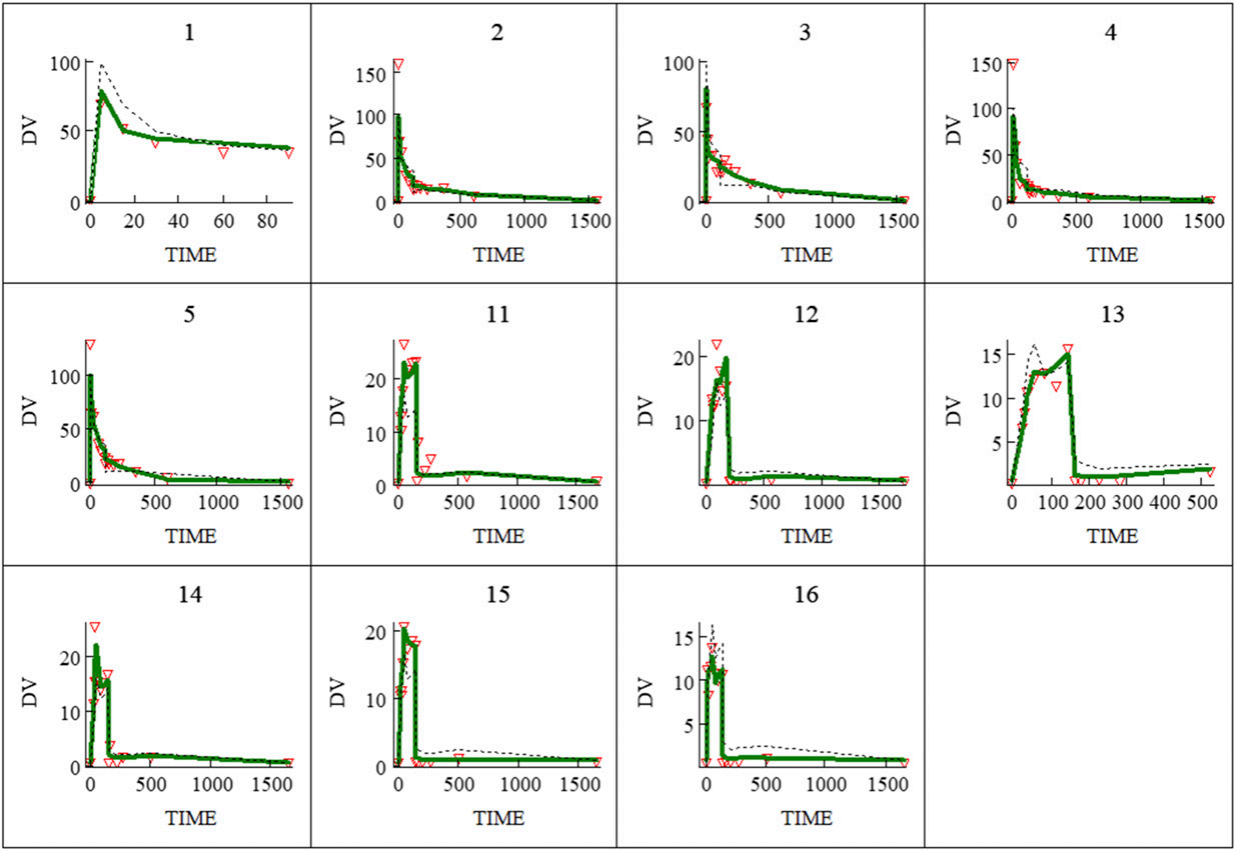
Plots of effect over time (minutes). Effect is the thromboelastographic response time (DV = R-time, minutes) Individual plots 1 to 5 are those sheep who did not undergo cardiopulmonary bypass. Individual plots of sheep 11 to 16 are those who underwent 120 min of cardiopulmonary bypass and who were administered idarucizumab for dabigatran reversal of effect. Idarucizumab effect was immediate and sustained. Observed concentrations are shown as inverted red triangles. Individual baysian estimates are shown as a solid green line. The population estimate is shown as a dashed black line.

Interaction between idarucizumab and dabigatran on the effect measure, R-time, was explained using the antagonist potency concentration (EA_50_) of 29.9 mg/L. The idarucizumab loading dose 0.25 mg/kg was very effective in those sheep who had undergone bypass, lowering R-times to baseline concentrations observed before the use of dabigatran. This effect was maintained for the duration of the study and is shown in individual plots in Figure 4.

## Discussion

We previously investigated the pharmacokinetics and pharmacodynamics of dabigatran administered to sheep.^5^ That information was used to predict the dose required to achieve a target concentration of 5 mg/L in sheep undergoing cardiopulmonary bypass; a concentration predicted to be associated with a specific target effect (R-time 16 min).^5^ The subsequent study of sheep undergoing cardiopulmonary bypass achieved a mean dabigatran concentration of 3.5 mg/L.^8^ The current analysis suggests this to be attributable to both a small increase in dabigatran clearance and a major expansion of the peripheral volume of distribution during bypass. Altered organ blood flow, temperature changes or inflammatory responses can affect clearance. Protein binding changes, hemodilution, and circuit drug adherence influence apparent volume of distribution.

Monitoring of R-time is a convenient point-of-care whole blood test that is rapidly achieved. The short equilibration half-time estimated (T_1/2_keo 0.011 min) is consistent with the drug’s immediate reversible binding to thrombin, inhibiting its activity. This makes R-time a coagulative measure useful for dabigatran dose adjustment. The need for subsequent monitoring after initiation of bypass is because the use of extracorporeal circuits are associated with additional covariates that can affect both pharmacokinetic (e.g., clearance, volume) and pharmacodynamic (E_MAX_, C_50_) parameter estimates. Hemodilution and inflammatory responses have further impact on the coagulation cascade. Sheep undergoing cardiopulmonary bypass had a lower EC_50_ than those not undergoing cardiopulmonary bypass. The impact of cardiopulmonary bypass and its effect on the coagulation cascade is demonstrated in Figure 3. Responses (R-times) are above the population mean value in those sheep with dabigatran concentrations smaller than 5 mg/L; the observed mean dabigatran concentration during bypass. Those rich data skew the concentration-response at higher concentrations.

The sheep bypass study^8^ used a bigger idarucizumab dose for reversal of dabigatran effect than the earlier non-bypass sheep study.^5^ This was because dabigatran rebound after neutralization of the dabigatran by idarucizumab has been reported in the literature, with higher concentrations predictive of larger rebound effect.^17^ The bigger dose provided data that enabled examination of the dabigatran- idarucizumab interaction. We were unable to assay idarucizumab concentration in the sheep bypass study^8^ because the bond binding the two drugs together is strong. Idarucizumab binds dabigatran with an affinity that is 350 times as high as that observed with thrombin.^18^ However we assumed that idarucizumab pharmacokinetics in sheep are similar to humans. This remains unknown, but it is of value to note that the pharmacokinetic parameter estimate for clearance of dabigatran in sheep was similar to that noted in adult humans.^19,20^

Dabigatran as an alternative to heparin has advantages and disadvantages. As a direct thrombin inhibitor, it is member of a class of drugs that broadly have the most successful application to anticoagulation for cardiopulmonary bypass. However, it is the only member of the class that can be easily and rapidly reversed by an FDA approved drug. While r-hirudin, argatroban and bivalirudin have all been used to maintain blood fluidity in human cardiopulmonary bypass, the lack of a reversal agent means all have been associated with excessive bleeding after surgery.^21–23^ In addition, it has been shown that argatroban, bivalirudin and lepirudin (r-hirudin) are inferior to heparin in preventing clot initiation and clot strength.^24^

An obvious limitation of dabigatran is that it is at a very early stage of development for this indication, and large investments in time and money will need to be made before it is ready for clinical use. Whether or not a company will be willing to make an investment in this drug as a usable clinical strategy is unknown, but this may be made more likely as the published knowledge of its utility increases. Dabigatran is primarily eliminated by renal clearance, so may appear to be unsuitable for use in patients with end-stage renal failure. However, the ability to completely reverse its effects with idarucizumab obviates this issue. Dabigatran-idarucizumab complexes depend much less on renal elimination with a significant degree of metabolism occurring through protein catabolism. It should be noted that the duration of effect of bivalirudin is greatly extended in patients with severe chronic kidney disease and is associated with problematic bleeding after cardiopulmonary bypass.^23^

Although data collection is this current sheep bypass study^8^ was richer (11 sheep, 233 observations) than the earlier non-bypass study (5 sheep, 135 observations),^5^ dabigatran concentrations after administration of idarucizumab were unavailable for interrogation. The two drugs bind together and individual drug concentrations were unobtainable. Further, the administration of idarucizumab alters the elimination kinetics of dabigatran. The elimination is changed from renal excretion of a relatively small molecule to less renal excretion with some protein catabolism of the larger drug-antibody complex. Prediction of dabigatran drug concentration after idarucizumab administration was possible based on earlier data from the study before and during cardiopulmonary bypass.

We have explored the sheep as an animal model because there is an increasing focus on rapidly translating findings in the laboratory to clinical applications in cardiovascular procedures. This has resulted in a shift from small to larger animal models, such as sheep, that closely correlate with human anatomy and physiology and allow use of commercially available bypass units used in humans. This current study informs future studies by defining dabigatran pharmacokinetics, defining a dabigatran concentration-response relationship and characterizing the dabigatran-idarucizumab pharmacodynamic response using a competitive interaction model. The current study also raises other areas of investigation such as explanation for the expansion of the peripheral volume of distribution,^25^ impact of the inflammatory response on thromboelastic measures, and characterization of idarucizumab pharmacokinetics both on and off cardiopulmonary bypass in sheep.

## Supplemental Material

Supplemental material - Dabigatran-idarucizumab Pharmacokinetics-pharmacodynamics in sheep undergoing cardiopulmonary bypass Supplemental material for TDabigatran-idarucizumab Pharmacokinetics-pharmacodynamics in sheep undergoing cardiopulmonary bypass by Michael P Eaton, Sergiy M Nadtochiy, Tatsiana Stefanos, and Brian J Anderson in Perfusion.

## Data Availability

The data that support the findings of this study are available from the corresponding author upon reasonable request.*
