# Supplementary material for: Dabigatran-idarucizumab pharmacokinetics-pharmacodynamics in sheep undergoing cardiopulmonary bypass
Source: Perfusion. 2025 Dec 1;41(5):658–64. doi: 10.1177/02676591251406086 (PMC13323899; doi:10.1177/02676591251406086)
Supplement: Supplemental material - Dabigatran-idarucizumab Pharmacokinetics-pharmacodynamics in sheep undergoing cardiopulmonary bypass [file sj-pdf-1-prf-10.1177_02676591251406086.pdf]

Supplementary NM-TRAN Code

```
$PROB Dabigatran CPB IDAPKPD

$ABBR DERIV2=NO

$INPUT ID TIME MDV DUR RATE AMT DVID CPB DV WT CMT IDA FLOW

$DATA Sheep_Dab_CPB_combo_PKPD.csv

IGNORE #

$ESTIM MAXEVAL=9999 NSIG=3 SIGL=9 PRINT=1 NOABORT METHOD=CONDITIONAL
INTERACTION

MSFO=dabi_2cmt_CPB.msf

$COV

; Pharmacokinetics Dabigatran

$THETA (0.01,0.0509,200)   ; POP_CL

$THETA (0.5,3.89,200)      ; POP_V1

$THETA (0.001,0.229,200)  ; POP_Q

$THETA (0.5,11.4,200)      ; POP_V2

;CPB device

$THETA (0,1.,) FIX        ; POP_Q3

$THETA (0,0.45,) FIX      ; POP_V3


;Factor for change in parameter after bypass start

$THETA (0,1.,) FIX  ; F_CL

$THETA (0,1.,) FIX  ; F_Q

$THETA (0,1.,) FIX ; F_V1

$THETA (0,2.25,)   ; F_V2


;RESIDUAL UNIDENTIFIED VARIABILITY

$THETA (0,0.00708,)  ; RUV_SDCP

$THETA (0,0.227,)  ; RUV_CVCP
```

```
$OMEGA BLOCK(4)
0.184 ; PPV_CL
0.0199 0.393 ; PPV_V1
-0.116 -0.219 0.483 ;PPV_Q
0.113 -0.00988 0.0175 0.134 ; PPV_V2

$OMEGA 0 FIX      ; PPV_Q3
$OMEGA 0 FIX      ; PPV_V3

; Pharmacokinetics Idarucimab (Wang Adv Ther 2020
$THETA (0.001,0.0394,200) FIX    ; POP_CLI
$THETA (0.5,6.47,200) FIX    ; POP_VI
$THETA (10,29.9, )       ; EA50

; Pharmacodynamics
$THETA (0.1,180., 200 ) FIX    ; EMAX
$THETA (0.1,1.,8) FIX   ; HILL
$THETA (0.001,40.8, 200)  ; C50
$THETA (0.1,0.584,1) FIX   ; E0
$THETA (0.001,0.011,200) FIX     ; TEQ

;RESIDUAL UNIDENTIFIED VARIABILITY
$THETA (0,0.119,)  ; RUV_SDPD
$THETA (0,0.535,) ; RUV_CVPD

$OMEGA 3.55     ; PPVEA
$OMEGA 0.0785    ; PPVC50
$OMEGA 0.0001 FIX   ; PPVEMX
```

```
$OMEGA 0 FIX     ; PPVE0
$OMEGA 0 FIX     ; PPVTEQ
$OMEGA 0 FIX     ; PPVHIL

;RESIDUAL UNIDENTIFIED VARIABILITY (OBSERVATIONS)
$OMEGA 0 FIX     ; PPV_RUVCP
$OMEGA 0 FIX     ; PPV_RUVPD

$SIGMA 1. FIX ; EPS1

$SUBR ADVAN13 TOL=9
$MODEL
 COMP (DIGAB)
 COMP (PERIPH)
 COMP (PUMP)
 COMP (EFFECT)
 COMP (IDA)

$PK
  IF (AMT.GT.0) DOSE=AMT

  IF (NEWIND.LE.1) THEN
       DOSE=0
       LN2=LOG(2)
   ENDIF

   FSZV=WT/70
   FSZCL=FSZV**0.75
   FSZT=FSZV**0.25
```

```
IF(CPB.EQ.1)THEN ; CPB on
 FCL=F_CL
 FQ=F_Q
 FV1=F_V1
 FV2=F_V2
ELSE ; CPB off
 FCL=1
 FQ=1
 FV1=1
 FV2=1
ENDIF

CL=FSZCL*POP_CL*FCL*EXP(PPV_CL)
Q=FSZCL*POP_Q*FQ*EXP(PPV_Q)
Q3=POP_Q3*FLOW*EXP(PPV_Q3)
V1=FSZV*POP_V1*FV1*EXP(PPV_V1)
V2=FSZV*POP_V2*FV2*EXP(PPV_V2)
V3=POP_V3*EXP(PPV_V3)

S1=V1
S2=V2
S3=V3
D1=DUR

EA50=EA50*EXP(PPVEA)
CLI=POP_CLI
VI=POP_VI
```

```
 E0=E0*EXP(PPVE0)
 C50=C50*EXP(PPVC50)
 EMAX=EMAX*EXP(PPVEMX)
 HILL=HILL*EXP(PPVHIL)
 TEQ=FSZT*TEQ*EXP(PPVTEQ)
 KEQ=LN2/TEQ


$DES
 DCP=A(1)/V1
 DC2=A(2)/V2
 DC3=A(3)/V3
 DCE=A(4)
 DCI=A(5)/VI

 DADT(1)= DC2*Q + DC3*Q3*CPB - DCP*(CL+Q+ (Q3*CPB))
 DADT(2)= Q*(DCP-DC2)
 DADT(3)= DCP*Q3*CPB - DC3*Q3*CPB
 DADT(4)= KEQ*(DCP-DCE)
 DADT(5)= - DCI*CLI

$ERROR
 CP=A(1)/V1
 APUMP=A(3)
 CPUMP=APUMP/V3
 CE=A(4)
 CI=A(5)/VI

"IF (CE.LE.0)CE=1D-10
```

```
 CEN=CE**HILL
 C50N=C50**HILL

  ANTAG=1+CI/EA50
  FX=E0 + EMAX*CEN/(C50N*ANTAG+CEN)

  PROP=CP*RUV_CVCP
  ADD=RUV_SDCP
  SDCP=SQRT(PROP*PROP + ADD*ADD)*EXP(PPV_RUVCP)

  PROPD=CE*RUV_CVPD
  ADDPD=RUV_SDPD
  SDPD=SQRT((PROPD*PROPD) + (ADDPD*ADDPD))*EXP(PPV_RUVPD)

 IF(DVID.LE.1) THEN  ;; DIGABACTRAN CONCENTRATION
  Y=CP + SDCP*EPS1
 ENDIF

IF(DVID.EQ.2) THEN ;; EFFECT COAG
 Y=FX + SDPD*EPS1
ENDIF

$TABLE ID TIME WT CL V1 Q V2 Y MDV DVID WT APUMP CPUMP
ONEHEADER NOPRINT FILE=dabi_2cmt.fit
```
